# Effects of the Fear of COVID-19 and Efficacy of Coping Behavior for Infectious Diseases after the End of COVID-19: Moderating Effects of Cyberchondria and eHealth Literacy

**DOI:** 10.3390/bs13080663

**Published:** 2023-08-08

**Authors:** Goo-Churl Jeong, Kunho Lee, Yinghua Jin

**Affiliations:** 1 Department of Counseling Psychology, College of Health and Welfare, Sahmyook University, Seoul 01795, Republic of Korea; gcjeong@syu.ac.kr (G.-C.J.); leekunho@syu.ac.kr (K.L.); 2 Department of Interdisciplinary Arts and Performance, Graduate School, Sahmyook University, Seoul 01795, Republic of Korea

**Keywords:** COVID-19 pandemic, fear, efficacy, infectious diseases, cyberchondria, eHealth literacy

## Abstract

As the coronavirus disease (COVID-19) pandemic ends, it is worth considering whether the ability to cope with such a pandemic has improved. The initial response to COVID-19 was hampered by the fear of new infectious diseases and spread of misinformation on the Internet. This highlights the need to enhance our ability to critically evaluate information rather than indiscriminately search for and trust information on the Internet. Therefore, this study examined how cyberchondria and eHealth literacy moderate the effect of fear of COVID-19 on the efficacy of coping behaviors for future epidemics and pandemics. The participants were 1000 adults in South Korea who were selected based on population proportionality. The results showed that fear of COVID-19 was significantly positively related to cyberchondria, and eHealth literacy was significantly positively related to the efficacy of coping behaviors. Further, the fear of COVID-19 had a significantly negative effect on the efficacy of coping behaviors, and the moderating effect of cyberchondria varied according to the level of eHealth literacy. These results emphasize the importance of eHealth literacy in enabling critical decision-making regarding misinformation.

## 1. Introduction

On 5 May 2023, the World Health Organization (WHO) declared the end of the global health emergency of COVID-19 [1]. With the end of the COVID-19 pandemic and the resulting chaos, it is imperative to reflect on the effects they have had and the ways forward. In particular, the danger of rapidly spreading infectious diseases must be recognized, along with the acknowledgement that infectious diseases have a significant impact on human survival and life. During this pandemic, COVID-19 spread rapidly before the new virus could be studied and a solution could be found. Consequently, searching the Internet for information about COVID-19 became common, and people have since been exposed to considerable misinformation. Therefore, the ability to critically engage with the correct information is crucial, especially considering the plethora of information on the Internet. Many people uncritically acquired misinformation about COVID-19 through the Internet, which further contributed to the spread of fear of COVID-19. Previous studies show that the fear of COVID-19 significantly deteriorates an individual’s mental health and sleep quality, and has an indirect negative effect on preventive behaviors [2]. Furthermore, the fear of COVID-19 significantly increased hopelessness [3] and reduced college students’ self-efficacy in career decision-making through depression [4]. These results suggest that the fear of COVID-19 is related to a decrease in hope for the future and a reduced sense of efficacy. The experience of the COVID-19 pandemic underscores the importance of an early response, as it is important to control the spread of an infectious disease before it becomes a pandemic. Therefore, when the outbreak of a new infectious disease occurs, it is imperative to recognize the signs of the infectious disease in oneself, take proper precautions against infectious diseases and effective epidemic control measures, and cooperate with the government’s initial response measures, such as social distancing [5]. As COVID-19 is likely not the last epidemic, assessing how proactively we can deal with a new epidemic or pandemic in the future is necessary. Therefore, we aimed to evaluate whether the fear of COVID-19 reduced the sense of efficacy in dealing effectively with the pandemic.

When one is unhealthy or symptomatic, it is common to search for health information on the Internet. According to the 2021 Internet Usage Survey conducted by the Korea Institute for Intelligent Information Society (NIA), the percentage of people who used the Internet to search for health information was 70.2% [6]; further, 37.5% of Internet users seeking health information users searched for information on a specific disease [7]. These results indicate that accessing health-related information on the Internet is prevalent in society; however, problems may arise if the health information is focused on rather than getting professional medical treatment. “Cyberchondria” is not simply searching for health information, but a tendency to excessively search online for medical information, which increases stress, as well as obsessively searching for information despite obstacles in daily life [8,9]. Obtaining health information on the Internet when one lacks the ability to choose the correct information adds to excessive concerns about health. A study reported that the longer the Internet usage time of college students, the lower the likelihood of performing preventive actions related to coughing or non-face-to-face exercises [10]; further, the more severe the overload of COVID-19 information, the greater the negative well-being [11]. In fact, in the early stages of the pandemic, high levels of misinformation led to several inappropriate responses to COVID-19 [12]. In situations involving a new infectious disease where information is lacking, building the capacity to critically accept accurate information from reliable institutions or sources is necessary.

Critical eHealth literacy refers to the cognitive ability to critically analyze and control information obtained through the Internet, and subsequently utilize it [13]. At the beginning of the pandemic, the WHO Director-General warned of the danger of misinformation through a notice on how to fight an infodemic and epidemic [12]. Health-related misinformation is among the most serious social problems because it is directly related to human life. Misinformation caused confusion about the truth [14], which led to anxiety and panic about the COVID-19 pandemic [15,16]. People may experience symptoms such as fatigue, stress, anger, and insomnia because of the prevalence of fake news [16,17,18]. Although not all fake news is spread intentionally, it is likely to thrive in the early stages of a new epidemic. We have already learned about the dangers of fake information during the COVID-19 pandemic; therefore, we must possess critical literacy capabilities to obtain health information when an infectious disease epidemic occurs. In a previous study examining Koreans’ identification with fake news (eight questions, maximum eight points), the overall mean score was 4.52 points, and those aged under 30 and over 60 years had lower than average scores [19]. These results indicate that only over half of the sample could identify fake news, suggesting that eHealth literacy education is urgently required. In a previous study compared with a group with low eHealth literacy, the group with high eHealth literacy had a probability of performing non-face-to-face exercises and preventive behaviors that was approximately 6.1 times higher, and a probability of performing distancing preventive behaviors that was 4.3 times higher [10]. Thus, appropriate coping behaviors can be encouraged through critical eHealth literacy. In summary, the fear of COVID-19 reduces the efficacy of coping behaviors in the event of an infectious disease epidemic, and cyberchondria regulates this relationship. However, we expected differences in the regulatory effects of cyberchondria based on the level of eHealth literacy.

**Figure 1:**
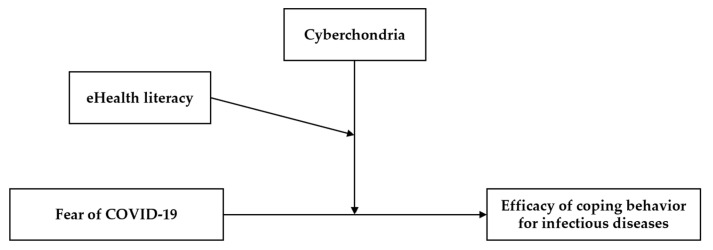
Research model.

This study aimed to verify the moderating effects of cyberchondria and eHealth literacy on the relationship between the fear of COVID-19 and efficacy of coping behaviors for infectious diseases. The model used in this study is illustrated in Figure 1.

The hypotheses of this study are as follows:

**Hypothesis 1.**  *Cyberchondria will have a moderating effect on the relationship between the fear of COVID-19 and efficacy of coping behaviors for infectious diseases; that is, more severe cyberchondria will strengthen the negative relationship between the fear of COVID-19 and coping efficacy.*

**Hypothesis 2.**  *eHealth literacy will moderate the moderating effect of cyberchondria on the relationship between the fear of COVID-19 and efficacy of coping behaviors for infectious diseases; that is, higher eHealth literacy will strengthen the moderating effect of cyberchondria.*

## 2. Materials and Methods

### 2.1. Participants

The participants were 1000 adults between the ages of 19 and 65 years, of which 507 were men (50.7%) and 493 were women (49.3%). The average age of the participants was 43.7 (SD = 12.7) years. Further, 354 (35.4%) participants had been infected with COVID-19 and 937 (93.7%) had completed the COVID-19 vaccination schedule.

### 2.2. Procedure

This study was approved by the Institutional Review Board of Sahmyook University (IRB No. 2022-10-003). We collected data through a professional survey agency from 29 February 2023 to 7 March 2023. For sampling the research participants, a population proportion sampling that considered participants’ sex, age, and residential area was used. Data were collected from 1000 individuals with a sampling error of 3.1%. We explained the study purpose in an explanatory note to the participants and designed it such that only those who voluntarily agreed to participate responded to the survey. Personal information was not collected and the scope of data use was limited to research purposes.

### 2.3. Instrument

#### 2.3.1. Fear of COVID-19

The fear of COVID-19 was measured using the Korean version of the Fear of COVID-19 Scale [20] developed by Ahorsu et al. [21]. The scale consisted of seven items that evaluated physiological responses (three items) and emotional responses (four items). The scores ranged from 1 to 5, with a higher total score indicating a greater fear of COVID-19. The Cronbach’s α for the scale was 0.82 in Ahorsu et al. [21], and 0.92 in the current study.

#### 2.3.2. Cyberchondria

The Korean version [22] of the Cyberchondria Severity Scale (CSS) developed by McElroy and Shevlin [23] and shortened to 15 items by Barke et al. [24] was used. The subfactors of the CSS were obsessive-compulsive, distressing, excessive, reassuring, and distrust of healthcare professionals. The scores ranged from 1 to 5, with higher scores indicating higher levels of online hypochondria. The Cronbach’s α for the Korean version of the scale was 0.82 [22], and 0.68 in the current study.

#### 2.3.3. eHealth Literacy

This study used the eHealth Literacy Scale developed by Lee [13]. It evaluated communicative, functional, and critical eHealth literacy factors. Additionally, the critical eHealth literacy scale was used in this study. It consisted of 12 items. Scores ranged from 1 to 5, with higher scores indicating that one is more skilled at finding and evaluating health information obtained on the Internet. The Cronbach’s α in a previous study was 0.93 [13], and 0.93 in this study.

#### 2.3.4. Efficacy of Coping Behaviors for Infectious Diseases

The Efficacy of Coping Behaviors for Infectious Diseases (ECBID) scale developed by our research team was used. The ECBID measured coping efficacy in relation to the COVID-19 pandemic. The researchers developed 18 items by referring to the infectious disease preventive behavior scale and organized them into nine items through reliability and construct validity tests. For construct validity analysis, 360 data points corresponding to 20 times the number of questions (18 items) were randomly extracted to form Sample 1; for confirmatory factor analysis, 300 non-redundant data points were randomly extracted, and Sample 2 was composed. An exploratory factor analysis was performed using Sample 1, and nine items were selected considering commonality (>0.30) and factor coefficients (>0.30). Using Sample 2, a confirmatory factor analysis was performed on nine questions to confirm the model fit and factor coefficients. Regarding the goodness of fit of the final model, the *χ*^2^ value was 28.39 (*df* = 27), which was not statistically significant (*p* = 0.391), and the standardized factor coefficient of the item was 0.55 or higher. The goodness-of-fit indices of the model—TLI = 0.998, CFI = 0.999, SRMR = 0.025, and RMSEA = 0.013 (90% CI = 0.000–0.048)—indicated that the confirmatory factor analysis model was appropriate. The following are some of the questions: When an epidemic occurs, “I can sensitively observe whether the characteristics of an infectious disease appear in me”, “I can carry out preventive actions against infectious diseases myself”, “I can follow the government’s initial response code of conduct”, and so on. The Cronbach’s α in this study was 0.88.

### 2.4. Data Analysis

The collected data were analyzed using IBM SPSS Statistics and IBM AMOS for Windows (version 25.0; IBM Corp., Armonk, NY, USA). We calculated the descriptive statistics of the study variables and presented the reliability of the scale by calculating the Cronbach’s α coefficient. Exploratory and confirmatory factor analyses were conducted to test the construct validity of the measurement tools and the correlation coefficients between the study variables. We analyzed the moderating effect using SPSS PROCESS program version 4.3 (Model 3) based on a hierarchical regression analysis [25]. To better understand the moderation effect, the levels of the moderator variables were plotted as (M + 1SD), medium (M), and low (M-1SD) centered on the mean.

## 3. Results

### 3.1. Descriptive Statistics of Research Variables

The means, standard deviations, skewness, and kurtosis of the research variables were calculated (Table 1). The absolute values of the skewness and kurtosis of the study variables were less than 1.32, indicating the normality of the research variables.

### 3.2. Correlation between Research Variables

Table 2 presents the results of correlation analyses of the research variables. Fear of COVID-19 was significantly positively correlated with cyberchondria and significantly negatively correlated with the efficacy of coping with infectious diseases. In addition, eHealth literacy was significantly and positively correlated with the efficacy of coping with infectious diseases.

### 3.3. Moderating Effect of Cyberchondria and eHealth Literacy

The moderating effect was analyzed using regression analysis in SPSS PROCESS (Model 3). To analyze the moderating effect, sex, age, and COVID-19 infection experience were used as control variables, and the moderating effect was analyzed by inserting an interaction term. The interaction terms were constructed by multiplying them after mean centering to aid interpretation.

Fear of COVID-19 had a significant negative effect on efficacy (*B* = −0.16, *p* < 0.001). The moderating effect of cyberchondria on the relationship between fear of COVID-19 and the efficacy of coping behaviors for infectious diseases was statistically significant (*B* = 0.13, *p* = 0.002). In other words, the higher the level of cyberchondria, the stronger the negative effect of the fear of COVID-19 on the efficacy of coping with infectious diseases. In the relationship between the fear of COVID-19 and efficacy of coping with infectious diseases, the moderating effect of cyberchondria was significant, thereby supporting Hypothesis 1.

**Figure 2:**
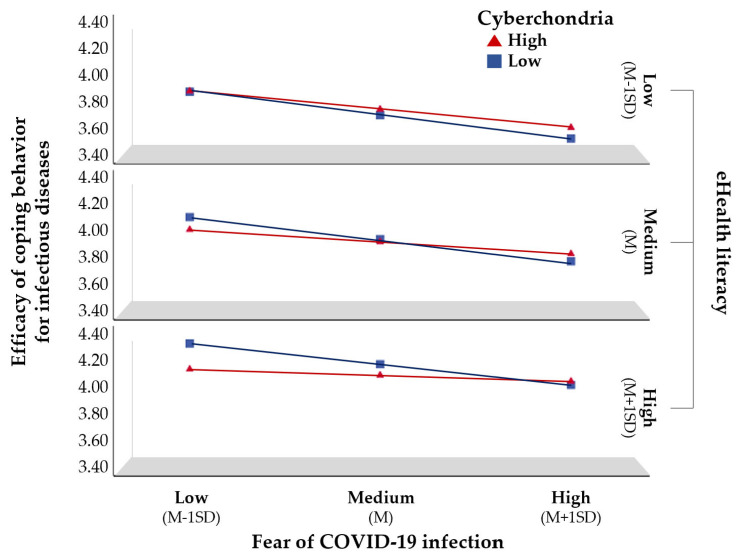
Moderating effects of cyberchondria and eHealth literacy.

To test Hypothesis 2, we analyzed whether the moderating effect of cyberchondria differed according to the level of critical eHealth literacy. The results of the analysis (Table 3) showed that the three-way interaction term was statistically significant (*B* = 0.12, *p* = 0.022), confirming the moderating effect of eHealth literacy. To understand this more clearly, the results are shown in Figure 2. The moderating effect of cyberchondria was weak at low levels of eHealth literacy; on the contrary, it was very strong at high levels of eHealth literacy. In other words, in groups with high eHealth literacy, the lower the cyberchondria, the stronger the negative effect of the fear of COVID-19 on the efficacy of coping behavior. However, in the group with low eHealth literacy, the negative effect of the fear of COVID-19 on the efficacy of coping behaviors for infectious diseases did not differ according to the level of cyberchondria. Further, eHealth literacy significantly moderated the moderating effect of cyberchondria on the relationship between the fear of COVID-19 and efficacy of coping behaviors for infectious diseases, thereby supporting Hypothesis 2.

## 4. Discussion

The COVID-19 pandemic is the most recent global outbreak of infectious disease, which has left many people with a fear of pandemics. Although COVID-19 does not have the highest mortality rate among infectious diseases, its rapid spread and lack of a cure are sufficient to generate fear. In this study, we found that a higher fear of COVID-19 was associated with significantly lower efficacy for coping behaviors for future emerging infectious diseases. These results are similar to those of previous studies showing that the fear of COVID-19 is positively related to depression [26] and negatively related to health attitudes [3] and coping behaviors [21]. Fear of the novel virus, along with a lack of accurate disease-related information, reduced coping efficacy in the absence of knowledge about appropriate preventive behaviors. Fear is also positively related to apathy, which is expected to reduce the efficacy of future coping behaviors. In addition, fear is closely related to decreased efficacy in future activities, including career decision efficacy among college students [5]. Therefore, we must prioritize addressing the excessive fear of COVID-19 in the past. Although COVID-19 has a high cumulative number of confirmed cases, its fatality rate is much lower than that of other coronavirus diseases, such as Severe Acute Respiratory Syndrome (SARS) and Middle East Respiratory Syndrome (MERS). As of 17 May 2023, the global mortality rate for COVID-19 was 0.91% [27], compared with 9.56% for SARS and 34.38% for MERS [28]. In South Korea, the COVID-19 mortality rate is 0.11% [29]. This demonstrates that during the outbreak, the Korean government, medical institutions, and related agencies actively collaborated to improve medical support and treatment systems, built a big data platform for infectious diseases, and rapidly developed a vaccine to respond effectively to COVID-19 [30]. Overcoming the COVID-19 pandemic has enhanced South Korea’s ability to respond effectively and appropriately to other pandemics and emerging infectious diseases. Therefore, although caution and vigilance are necessary in the event of an outbreak, there is no need to spread undue fear.

Excessive anxiety regarding health can be overwhelming, particularly in the face of an emerging infectious disease. In the present study, lower cyberchondria moderated the negative effect of the fear of COVID-19 on coping efficacy; that is, lower cyberchondria led to greater improvements in coping efficacy when the fear of COVID-19 was lower. This suggests that while it is important to primarily reduce the fear of COVID-19, excessive preoccupation with the pandemic and indiscriminate information-seeking behaviors do not appear to significantly increase coping efficacy, even when the fear reduces. The recent development of devices, such as smartphones, has dramatically increased the accessibility of online information. Previous studies show that people trust and are influenced by health information on social media [31]. Misinformation can disrupt positive health behaviors and lead to highly sensitive social anxieties, such as violence, distrust, social disruption, and attacks on healthcare professionals. Cyberchondria can magnify individual anxiety and prevent individuals from effectively coping with health anxiety [32,33]; in addition, indiscriminate online information-seeking has been linked to negative outcomes in coping with illness [34]. These are in line with our findings.

When analyzing the three-way interaction term between the fear of COVID-19, cyberchondria, and eHealth literacy, we found that the moderating effect of cyberchondria was not significant when eHealth literacy was low, whereas it was stronger when eHealth literacy was high. In other words, the negative effect of the fear of COVID-19 on coping efficacy was stronger when cyberchondria was low in the high eHealth literacy group. In the high eHealth literacy and low cyberchondria groups, coping efficacy increased as the fear of COVID-19 decreased. Those with high eHealth literacy and high levels of cyberchondria did not show a significant decrease in coping efficacy as a function of fear. This is likely because critical eHealth literacy leads to more information searching because of cyberchondria, as well as the ability to critically judge whether the information is appropriate and accurate. Indeed, eHealth literacy has been shown to increase infection control awareness [35] and health promotion behaviors [36] through the seeking of accurate and reliable health information [37]. Our results are similar to previous findings showing that college students with higher levels of eHealth literacy were more likely to participate in quarantine and vaccination [38] and were more effective in coping with COVID-19 through preventive behaviors [39,40]. Developing the ability to read and evaluate information critically is important for appropriately responding to emerging infectious diseases. Therefore, searching for large amounts of information out of fear for one’s health should be avoided, and one should be able to critically analyze and accept reliable information. The study results warn us about the severity of COVID-19 misinformation and highlight the role of critical literacy skills in health-related information.

This study has some limitations. First, it was conducted in South Korea using stratified random sampling, which is appropriate for reflecting the local situation in Korea but limits the generalizability of the findings to other countries. In addition, the level of coping efficacy and fear of COVID-19 varies by country. Therefore, it is necessary to analyze and promote the efficacy of coping behaviors for new infectious diseases through research in other countries. Second, there are different types of eHealth literacy and the types and sources of Internet information are diverse. This study did not measure eHealth literacy in a way that considers all these factors, and there are limitations to expanding the interpretation of critical literacy skills to include all online health information. Third, this study was conducted among adults; however, it is necessary to conduct studies among adolescents, who are likely to live through future epidemics. Another limitation is that the study did not include elderly participants, who may have had limited access to the Internet. Despite these limitations, the study results emphasize the role and importance of eHealth literacy.

## 5. Conclusions

In this study, we found that a reduction in the fear of COVID-19 improved the efficacy of coping with infectious diseases among Korean adults. The moderating effect of cyberchondria varied according to the level of eHealth literacy. Therefore, to improve the efficacy of coping behavior for future infectious diseases, reducing the fear of COVID-19 is necessary. Now that the COVID-19 has officially ended, there is an opportunity to actively develop eHealth literacy skills and reduce indiscriminate searching for and relying on information on the Internet. Governments should keep communication channels open and provide reliable information, and citizens should trust the information provided by government agencies and actively participate in the suggested preventive actions. Populations vulnerable to emerging infectious diseases, especially multicultural families, should be identified and provided with critical literacy training, and such training programs should be actively implemented to reduce the incidence of cyberchondria.

## Figures and Tables

**Table 1:** Descriptive statistics for the research variables (*N* = 1000).

Variables	M ± SD	Skewness	Kurtosis
Fear of COVID-19	2.09 ± 0.78	0.65	0.02
Cyberchondria	2.94 ± 0.37	−0.04	0.54
eHealth literacy	3.60 ± 0.60	−0.38	0.42
ECBID	3.92 ± 0.51	−0.47	1.32

ECBID = Efficacy of Coping Behaviors for Infectious Diseases.

**Table 2:** Correlation coefficient between the research variables (*N* = 1000).

Variables	Fear of COVID-19	Cyberchondria	eHealth Literacy
Cyberchondria	0.36 ***		
eHealth literacy	−0.06	0.15 ***	
ECBID	−0.24 ***	−0.07 *	0.42 ***

ECBID = Efficacy of Coping Behaviors for Infectious Diseases. * *p* < 0.05, *** *p* < 0.001.

**Table 3:** Moderating effect test for the efficacy of coping behaviors for infectious diseases (*N* = 1000).

Variables	*B*	*SE*	*t*	*p*
Fear of COVID-19 ^(a)^	−0.16	0.02	−7.74	<0.001
Cyberchondria ^(b)^	−0.06	0.04	−1.43	0.151
Interaction 1 * ^(a × b)^	0.13	0.04	3.01	0.002
eHealth literacy ^(c)^	0.32	0.02	12.97	<0.001
Interaction 2 * ^(a × c)^	0.05	0.02	1.84	0.064
Interaction 3 * ^(b × c)^	−0.09	0.05	−1.75	0.080
Interaction 4 * ^(a × b × c)^	0.12	0.05	2.28	0.022
Sex	0.16	0.02	5.62	<0.001
Age	0.002	0.001	2.12	0.034
COVID-19 infection experience	0.01	0.02	0.65	0.515
*R* ^2^	0.271 (*F* = 36.93, *p* < 0.001)			

^a,b,c^ Letters indicate each research variable. * The interaction term is the product of mean-centered variables.

## Data Availability

The data presented in this study are not available elsewhere.
